# Fabrication of Network Spherical α-Al_2_O_3_ and Its Application on the Separator of Lithium-Ion Batteries

**DOI:** 10.3390/ma18030660

**Published:** 2025-02-02

**Authors:** Haiyang Chen, Huifang Zhang, Hongliang Huang, Mingjie Guo, Jiale Wang, Peng Wang, Bin Li, Junhong Chen

**Affiliations:** 1 School of Civil and Engineering, Hebei University of Architecture, Zhangjiakou 075000, China; chenhaiyang_123@163.com (H.C.); zhfdayanjing09@163.com (H.Z.); huanghong.5055@163.com (H.H.); 15832418063@163.com (M.G.); 19556374484@163.com (J.W.); wp2242@hebiace.edu.cn (P.W.); 2 Hebei Key Laboratory of Diagnosis, Reconstruction and Anti-Disaster of Civil Engineering, Zhangjiakou 075000, China; 3 Hebei Provincial Laboratory of Inorganic Nonmetallic Materials, Tangshan 063000, China; 4 School of Materials Science and Engineering, University of Science and Technology Beijing, Beijing 100083, China

**Keywords:** ceramic-coated separator, network spherical α-Al_2_O_3_, thermal stability, ionic conductivity

## Abstract

Ceramic-coated polyolefin separator technology is considered a simple and effective method for the improvement of lithium-ion battery (LIB) safety. However, the characteristics of ceramic powder can adversely affect the surface structure and ion conductivity of the separators. Therefore, it is crucial to develop a ceramic powder that not only improves the thermal stability of the separators but also enhances ion conductivity. Herein, network spherical α-Al_2_O_3_ (N-Al_2_O_3_) with a multi-dimensional network pore structure was constructed. Furthermore, N-Al_2_O_3_ was applied as a coating to one side of polyethylene (PE) separators, resulting in N-Al_2_O_3_-PE separators that exhibit superior thermal stability and enhanced wettability with liquid electrolytes. Notably, the N-Al_2_O_3_-PE separators demonstrated exceptional ionic conductivity (0.632 mS cm^−1^), attributed to the internal multi-dimensional network pore structures of N-Al_2_O_3_, which facilitated an interconnected and efficient “highway” for the transport of Li^+^ ions. As a consequence, LiCoO_2_/Li half batteries equipped with these N-Al_2_O_3_-PE separators showcased remarkable rate and cycling performance. Particularly at high current densities, their discharge capacity and capacity retention rate significantly outperformed those of conventional PE separators.

## 1. Introduction

Lithium-ion batteries (LIBs), owing to superior energy density and outstanding cycle life, are ubiquitous in electronic devices such as mobile phones and laptop computers, etc. [1,2,3,4]. In addition to these small-scale electronic devices, LIBs are also considered one of the most promising power sources for large-scale battery applications, including electric vehicles and energy storage systems, under the premise of ensuring cost and safety performance [1,5,6,7,8]. As one of the main components of LIBs, the separators facilitate the transport of lithium ions between the two electrodes while preventing direct contact between the cathode and anode, thereby playing a crucial role in determining the safety performance of LIBs [3,9,10]. At present, commercial separators utilized in LIBs are mainly polypropylene (PP), polyethylene (PE), and PP/PE multilayer porous membranes, owing to their excellent mechanical strength, superior electrochemical stability, uniform pore structure, and cost-effectiveness [5,9,11]. However, these separators suffer from poor thermal stability at an elevated temperature and a large polarity difference with electrolytes, thereby bringing additional troubles to the safety and electrochemical performance of LIBs during their application [4,5,12,13,14]. Therefore, improving the thermal stability and hydrophilicity of these polyolefin separators is critical to ensuring the safety performance of LIBs.

Currently, many strategies have been tried to improve the thermal stability and hydrophilicity of the separator, including the application of solid or gel electrolytes, the use of a separator made of bio-based materials, and the coating of ceramic powders on separators [5,14,15,16,17,18,19,20]. Among them, coating ceramic powder onto the polyolefin separator was recognized as an attractive approach because of its simple process and cost-effectiveness. For example, Chen et al. [19] integrated TiO_2_ onto PP separators by coupling plasma activation and atomic layer deposition. The results suggested that the modified PP separators demonstrated remarkable dimensional stability at high temperatures and a high ion migration number. Furthermore, the battery assembled with modified PP separators exhibited a higher rate of discharge capacity. Cho and co-authors [20] synthesized amino-functionalized SiO_2_ (N-SiO_2_) nanoparticles and coated them on both sides of the PE separators. The N-SiO_2_-coated PE separators displayed high thermal stability and good electrolyte wettability. Correspondingly, the battery with an N-SiO_2_-coated PE separator also presented high cycle stability and safety performance. Compared to ceramic powders such as TiO_2_ and SiO_2_, α-Al_2_O_3,_ with its outstanding performance, including high-temperature resistance, acid-alkali resistance and relatively low cost, has gained widespread recognition and extensive reporting in separator coatings [14,21,22,23]. However, the introduction of the ceramic coating increases the thickness of the separators, which in turn leads to a longer migration distance of Li^+^ ions, thus hindering their efficient transport within LIBs. Hence, enhancing the thermal stability of the existing PE separators while concurrently improving ion transport has remained a persistent challenge in the pursuit of developing safe and sustainable LIBs.

To our knowledge, the reason ceramic coatings impede the transport of Li^+^ ions, beyond the increase in thickness, is that Li^+^ ions can only traverse through the narrow gaps existing between the ceramic particles. Inspired by this, if α-Al_2_O_3_ particles applied on the separators possess a multi-dimensional network pore structure internally, they can not only enhance the transmission channels for Li^+^ ions but also circumvent the elongation of the transmission path that would otherwise occur when Li^+^ ions bypass the α-Al_2_O_3_ particles. Herein, network spherical α-Al_2_O_3_ (N-Al_2_O_3_) featuring a multi-dimensional continuous pore structure was crafted and applied as a coating on one side of the PE separators, yielding the N-Al_2_O_3_-PE separators. The results indicated that the N-Al_2_O_3_-PE separators displayed outstanding thermal stability, mechanical properties, and good compatibility with liquid electrolytes. Moreover, due to the internal multi-dimensional network pore structures of N-Al_2_O_3_, which provided an interconnected and efficient “highway” for Li^+^ ion transportation, the N-Al_2_O_3_-PE separators demonstrated outstanding ionic conductivity. Consequently, LiCoO_2_/Li half batteries equipped with these N-Al_2_O_3_-PE separators showed remarkable rate and cycle performance.

## 2. Experimental Section

### 2.1. Raw Materials

The main raw materials involved in the preparation of N-Al_2_O_3_ were as follows: sodium aluminate (NaAlO_2_, analytically pure, Beijing Innochem Technology Co., Ltd., Beijing, China), urea (analytically pure, Beijing Honghu Lianhe Chemical Products Co., Ltd., Beijing, China), and cetyl trimethyl ammonium bromide (CTAB, analytically pure, Sinopharm chemical reagent Co., Ltd., Shanghai, China). The preparation of the separators and the testing of their properties mainly contained the following feed stocks: PE separator (thickness 16 μm, Taobao Network, Hangzhou, China), poly(vinylidene fluoride) (PVDF, >99% pure, Beijing Honghu Lianhe Chemical Products Co., Ltd., Beijing, China), N-methyl-2-pyrrolidone (NMP, >99.5% pure, Beijing Honghu Lianhe Chemical Products Co., Ltd., Beijing, China), Tris buffer solution (pH 8.5, Taobao Network, Hangzhou, China), absolute ethyl alcohol (>99.8% pure, Sinopharm chemical reagent Co., Ltd., Shanghai, China), n-butanol (>99.9% pure, Beijing Honghu Lianhe Chemical Products Co., Ltd., Beijing, China), lithium cobalt oxides (LiCoO_2_, battery grade, Lizhiyuan Battery Materials Co., Ltd., Huzhou, China), acetylene black (technical grade, Lizhiyuan Battery Materials Co., Ltd., Huzhou, China), lithium tablet (battery grade, Lizhiyuan Battery Materials Co., Ltd., Huzhou, China), and 1 M LiPF_6_ in ethylene carbonate (EC)/dimethyl carbonate (DMC) mixtures (battery grade, Taobao Network, Hangzhou, China). The deionized water used in the above experiments was homemade in the laboratory. The α-A_l2_O_3_ used for comparison was purchased from Shandong Aluminum Industry Co., Ltd. (Weifang, China).

### 2.2. Preparation of N-Al_2_O_3_ Powder

Referring to our previous work [24], a N-Al_2_O_3_ precursor was synthesized by a hydrothermal method, using NaAlO_2_ and urea as raw materials without surfactants. In this paper, to prepare N-Al_2_O_3_ with a smaller size, we explored the influence of CTAB content and hydrothermal time on the size of its precursor. Firstly, NaAlO_2_ (4 g), urea (24 g), and different amounts of CTAB (0.1 g, 0.2 g, 0.3 g, and 0.5 g) were dissolved in 100 mL of deionized water and stirred for 30 min. The mixture was then transferred to a stainless steel reactor lined with Teflon, sealed, and placed in an oven at 180 °C for different incubation times (1 h, 1.5 h, 2 h, and 3 h). Once the temperature dropped to room temperature, the white sediment obtained by suction filtration was washed with deionized water and ethanol before being dried in the oven. For clarity, the resulting white sediment was labeled as Sx–y, where x represented the content of CTAB and y represented the holding time. Finally, the samples were sintered in a box furnace at 1300 °C for 3 h to obtain N-Al_2_O_3_.

### 2.3. Preparation of N-Al_2_O_3_-Coated PE Separator

Firstly, a uniform coating slurry was configured by mixing 1 g N-Al_2_O_3_ and 0.1 g PVDF in 5 mL NMP for some time with a magnetic stirrer at 500 rpm/min. The resulting slurry was then poured on one side of the PE separator and coated with a scraper blade to prepare the N-Al_2_O_3_-PE separators. Subsequently, the N-Al_2_O_3_-PE separators were left at room temperature for 1 h and then placed in a vacuum drying oven at 60 °C for 12 h.

### 2.4. Characterization

Field emission scanning electron microscopy (SEM, Nova Nano 450, FEI, Hillsboro, USA) and focused ion beam scanning electron microscopy (FIB-SEM, S9000X, TESCAN, Brno, Czech Republic) were applied to observe the microstructure of the as-obtained samples. Phase composition analysis was taken with an X-ray diffractometer (XRD, D/max2500, Rigaku Industrial Corporation, Tokyo, Japan) under the following conditions: voltage of 40 kV; current of 40 mA; Cu Kα radiation, step of 0.02°; scanning rate of 8°/min; scanning scope of 10–90°. The variation curve of Zeta potential with the pH value was recorded by a Zeta potential analyzer with a NANO-Z machine (Malvern Instruments LTD, Malvern, UK). The stress–strain curves of separators were collected by a universal testing machine operated at a strain rate of 20 mm/min. Due to the toxicity of liquid electrolytes and a similar polarity of water and liquid electrolytes, a DSA100 droplet shape analyzer was used to evaluate the affinity between separators and liquid electrolytes by measuring the static contact angle of water and separators. The high-temperature dimensional stability was determined by the contraction of separators treated at different temperatures (25, 130, 140, 150, and 160 °C) and holding this for 0.5 h. To further verify the thermal stability of separators, differential scanning calorimetry (DSC) curves of separators were recorded with a STA2500 TG-DTA/DSC synchrometer (NETZSCH-Geratebau GmbH, Bavaria, Germany) under a nitrogen atmosphere at a temperature rise rate of 10 °C/min. The electrolyte uptake (EU) of separators was characterized by the percentage of mass change in the separators soaking in the electrolyte for 2 h, which can be obtained by Formula (1), where *W*_0_ and *W*_1_ represent the weights of the separator before and after taking in the liquid electrolyte:

(1) $$EU(\%)=\frac{W_{1}-W_{0}}{W_{0}}\times 100\%$$

The porosity of separators was expressed by the ratio of the volume of n-butanol absorbed by the separators after immersing in n-butanol for 2 h to the volume of separators, which can be determined by Formula (2), where ∆*m* indicates the weight of absorbed n-butanol after separators being dipped in n-butanol, *ρ* represents the density of the n-butanol, and *V*_0_ stands for the volume of separators.

(2) P(%)=(Δmρ)V0×100%

The batteries employed in evaluating the electrochemical performance were assembled in a glove box filled with Ar, in which the contents of H_2_O and O_2_ were less than 0.01 ppm. The electrochemical impedance spectroscopy (EIS) of batteries assembled by different separators between two stainless steel plates was measured by an electrochemical working station (SI 1287/SI1260, Solartron Metrology) in the frequency range of 0.1 to 100 kHz with an amplitude of 5 mV to obtain the bulk resistance, and then the ionic conductivity of separators was calculated by Formula (3), where *σ* denotes the ionic conductivity, *d* indicates the thickness of the separator, and *R_b_* and *S* represent the bulk resistance and the area of the stainless steel electrode.

(3)  σ=dRbS

The interface compatibility between the separator and electrode was characterized by measuring the EIS of Li/Li symmetric batteries with various separators by the electrochemical workstation. The electrochemical stability of separators was investigated by linear sweep voltammetry (LSV) using Li/SS batteries using a Princeton multi-channel electrochemical workstation with a potential range of 2–6 V and a scanning rate of 0.1 mV/s. To test the rate performance and cycle stability of the batteries with different separators, 2025-type coin half batteries with Li metal as the anode and LiCoO_2_ as the cathode were built. The cathode material was prepared by coating a uniform slurry composed of LiCoO_2_, carbon black, and PVDF in a mass ratio of 90:5:5 and an appropriate amount of NMP as a solvent on aluminum foil. The electrode thickness and loading level (thickness of 50 μm, loading level of 1.75 mg cm^−2^) were controlled by a gap control rolling machine to roll the cathode material. Before the rate and cycling performance tests, the battery used was activated for 3 cycles at a current density of 0.2 C at room temperature after standing for 6 h. Then, the rate performance and cycling performance of these half cells (LiCoO_2_/Li) were evaluated in the LAND CT2001A battery testing system. A series of different charge and discharge current densities (0.2–10 C) were selected and tested over a voltage range of 3.0–4.2 V for the rate performance. The cyclic performance test was conducted at a current density of 1 C. The safety of separators in the battery system was to place the LiCoO_2_/Li half battery, fully charged to 4.2 V, at room temperature in a vacuum drying oven at 140 °C and used an electrochemical workstation to record its open circuit voltage (OCV).

## 3. Results and Discussion

**Figure 1:**
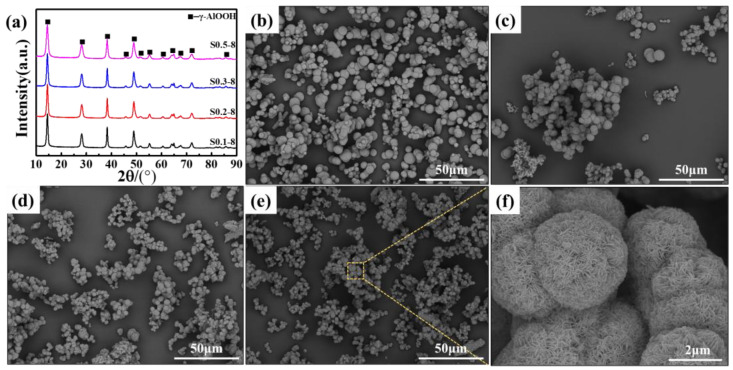
(**a**) XRD patterns of Sx–8, SEM images of (**b**) S0.1–8, (**c**) S0.2–8, (**d**) S0.3–8, and (**e**,**f**) S0.5–8.

Due to the adverse effects of the larger α-Al_2_O_3_ particle size on the porosity and thermal shrinkage of ceramic-coated separators, and given that the size of α-Al_2_O_3_ primarily depends on the size of its precursor, controlling the precursor size is of the utmost importance. Figure 1 presents the XRD patterns and SEM images of Sx–8. As shown in Figure 1a, the diffraction peaks exhibited by Sx-8 were all assigned to γ-AlOOH, and no peaks of other phases were detected, which was consistent with our previous reports. Figure 1b–f illustrate SEM images of Sx–8. It can be found that the morphologies of these samples were quite similar, all exhibiting a spherical shape. Upon closer examination, the particle size of Sx-8 demonstrated a pronounced decreasing trend as the amount of CTAB gradually increased. Table 1 presents the average particle size after statistical analysis, and the specific distribution is shown in Figure S1. When the content of CTAB increased to 0.5 g, the average particle size of the α-Al_2_O_3_ precursor decreased from 4.75 ± 1.64 μm to 2.64 ± 0.64 μm. As is well known, the formation of α-Al_2_O_3_ can be divided into two processes: nucleation and growth [25]. Among them, the nucleation rate, especially blasting nucleation, is very critical to the control of grain size [26]. Blasting nucleation can inhibit subsequent nucleation so that all grains have almost the same growth history. Additionally, blasting nucleation will consume a large amount of solute, resulting in a reduction in the amount of substrate required for crystal growth, thereby reducing the size of the as-obtained sample. It was reported that the rate of crystal nucleation mainly depended on supersaturation, temperature, and surface free energy [26]. The addition of CTAB reduced the surface free energy of the solution, thus speeding up the nucleation rate, so the average size of the α-Al_2_O_3_ precursor presented a decreasing trend.

**Figure 2:**
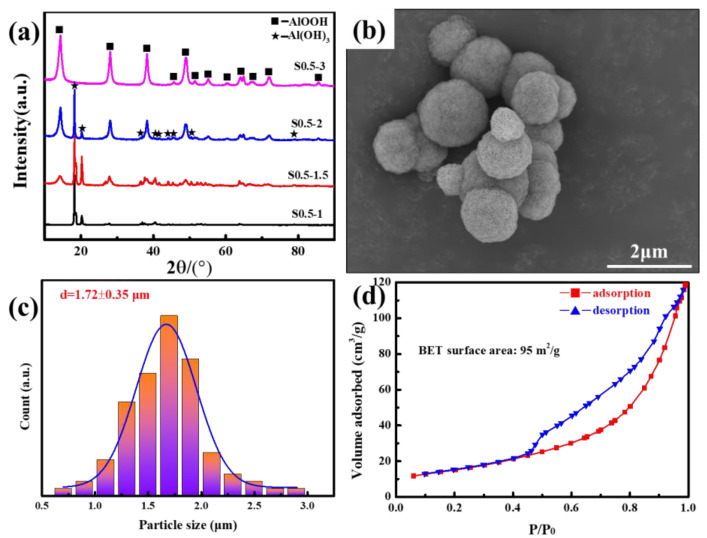
(**a**) XRD patterns of S0.5–y, (**b**) SEM images of S0.5–3, (**c**) size distribution of S0.5–3, and (**d**) nitrogen (N_2_) adsorption–desorption isotherms of S0.5–3.

Hydrothermal time is also a critical factor influencing the size of the α-Al_2_O_3_ precursor. It is widely recognized that as the hydrothermal time increases, the size of the particles obtained tends to grow larger due to the process of Ostwald ripening. The XRD pattern, as presented in Figure 2a, demonstrates the phase transition of S0.5-y at various holding times. At 1 h, only Al(OH)_3_ was detected. When the holding time was increased to 1.5 h, part of Al(OH)_3_ dissolved and precipitated to form γ-AlOOH. By 3 h, Al(OH)_3_ had completely dissolved, and pure γ-AlOOH was obtained. Regarding its morphology, S0.5–3 completely inherited the three-dimensional spherical morphology of S0.5–8 (Figure 2b). At the same time, its average size was also reduced from 2.64 ± 0.64 μm of S0.5–8 to 1.72 ± 0.35 μm (Figure 2c). The N_2_ adsorption–desorption curves of S0.5–3, shown in Figure 2d, exhibited a typical type IV isotherm feature, and an obvious H1 type hysteresis loop can be observed in the region where the relative pressure P/P_0_ was greater than 0.40. BET results indicated that the specific surface area of S0.5–3 was 95 m^2^/g.

**Figure 3:**
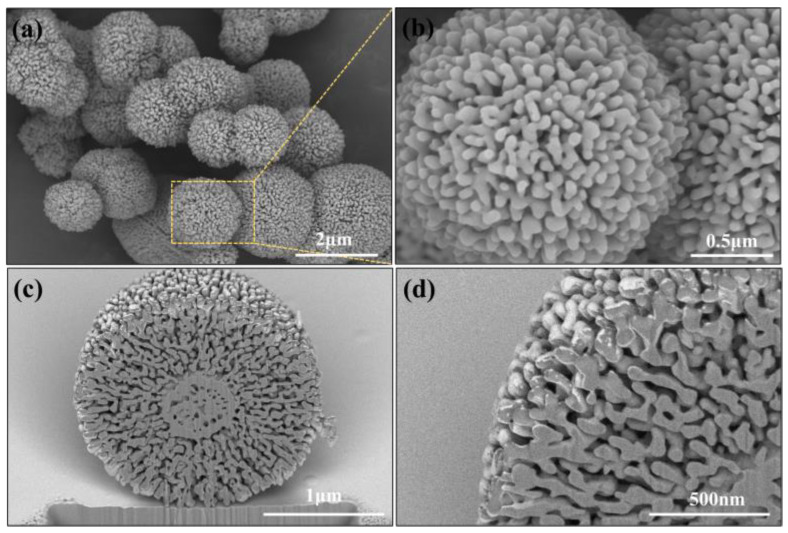
SEM images of (**a**,**b**) overall morphology and (**c**,**d**) cross-section of α-Al_2_O_3_ obtained through sintering S0.5–3.

The complete morphology and internal detailed structure of the α-Al_2_O_3_ obtained through sintering S0.5–3 are shown in Figure 3. Figure 3a demonstrates that the as-obtained α-Al_2_O_3_ maintained their perfect three-dimensional sphere after heat treatment, and no collapse or discontinuity was found on the sphere. Upon further observation of the magnified image, as shown in Figure 3b, the nanosheets on the surface of the original α-Al_2_O_3_ precursor were sintered into nanoparticles, with noticeable gaps between the nanoparticles. To observe the internal structure characteristics of the obtained α-Al_2_O_3_, the SEM observation of the cross-section of the synthesized α-Al_2_O_3_ was taken by FIB-SEM. As displayed in Figure 3c,d, the interior of the synthesized α-Al_2_O_3_ was covered with pores of tens to hundreds of nanometers in diameter, and even the pores were interpenetrating with each other to form a continuous network structure, which also demonstrated that N-Al_2_O_3_ had been successfully synthesized.

**Figure 4:**
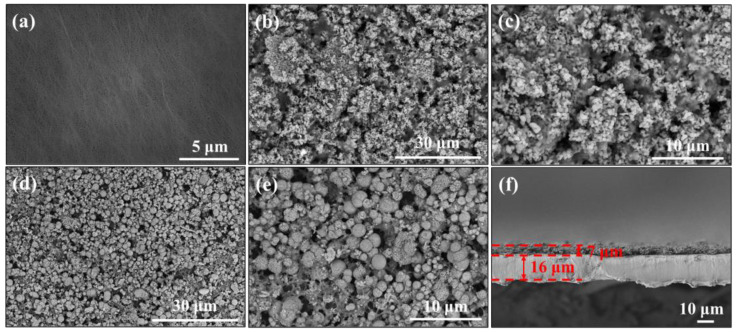
SEM images of (**a**) PE, (**b**,**c**) α-Al_2_O_3_-PE, and (**d**,**e**) N-Al_2_O_3_-PE separators; (**f**) the thickness of N-Al_2_O_3_-PE separators.

The surface features of the PE, α-Al_2_O_3_-PE, and N-Al_2_O_3_-PE separator are displayed in Figure 4a–f. The PE separator presented the same morphology as previously reported, featuring elliptical nanopores (Figure 4a) [15]. As for the α-Al_2_O_3_-PE and N-Al_2_O_3_-PE separators (Figure 4b–e), it can be seen that both α-Al_2_O_3_ and N-Al_2_O_3_ cover the surface of the separators. However, in comparison, there was still a small area of the PE separators in the N-Al_2_O_3_-PE separators that was not completely covered, primarily originating from the larger particle size of N-Al_2_O_3_. The thickness of α-Al_2_O_3_-PE and N-Al_2_O_3_-PE separators was consistently measured at 23 μm using a micrometer (Nanjing Halian Tools Co., LTD, Nanjing, China), which was further confirmed by examining the cross-section of the N-Al_2_O_3_-PE separators, as displayed in Figure 4f. Simultaneously, the weight of the PE separators modified by α-Al_2_O_3_ and N-Al_2_O_3_ increased by 0.45 mg/cm^2^ and 0.39 mg/cm^2^, respectively, in contrast to PE separators.

**Figure 5:**
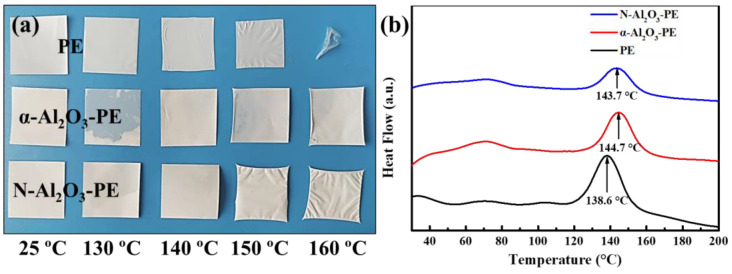
(**a**) Thermal shrinkage of various separators at different temperatures, and (**b**) the DSC curve of various separators.

The high-temperature dimensional stability of the separator is crucial for ensuring the safety performance of LIBs. To evaluate the influence of N-Al_2_O_3_ coating on the thermal stability of the separator, the thermal shrinkage of separators was investigated by observing the size changes in the separator after treatment at temperatures of 25 °C, 130 °C, 140 °C, 150 °C, and 160 °C for 0.5 h. As displayed in Figure 5a, at 130 °C, the PE separators experienced a weak contraction, while the α-Al_2_O_3_-PE and N-Al_2_O_3_-PE separators maintained their original shape. As for the temperature increasing to 140 °C and 150 °C, the PE separators demonstrated more obvious contraction. However, the α-Al_2_O_3_-PE and N-Al_2_O_3_-PE separators only showed a weak contraction at 150 °C. At 160 °C, the PE separators completely turned into a transparent form; the α-Al_2_O_3_-PE and N-Al_2_O_3_-PE separators also began to display a significant shrinkage. Based on these observations, it can be concluded that both α-Al_2_O_3_ and N-Al_2_O_3_ can improve the thermal stability of the PE separator. Relatively speaking, at the same temperature, especially at 150 °C and 160 °C, α-Al_2_O_3_ provided better protection to the separator than N-Al_2_O_3_. This result was related to the fact that the larger particle size of N-Al_2_O_3_ mentioned above did not fully cover the surface of the separator. To further assess their thermal stability, the DSC curves of these separators were recorded, as shown in Figure 5b. The exothermic peaks for the PE, α-Al_2_O_3_-PE, and N-Al_2_O_3_-PE separators were observed at 138.6 °C, 144.7 °C, and 143.7 °C, respectively, which aligned with the results observed in their thermal shrinkage tests.

**Figure 6:**
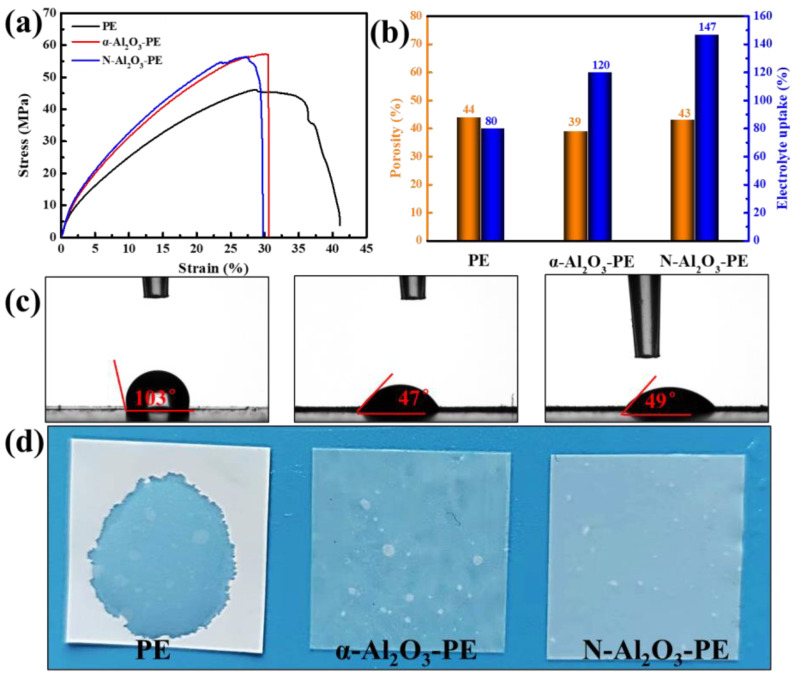
(**a**) Stress–strain curves, (**b**) porosity and electrolyte uptake, (**c**) photographs of contact angle value of various separators, and (**d**) photographs of the liquid electrolyte dropped onto various separators.

The excellent mechanical properties of the separators indeed hold great significance for their various applications, particularly in battery assembly as well as during the charge and discharge processes. Figure 6a depicts the stress–strain curves for the PE, α-Al_2_O_3_-PE, and N-Al_2_O_3_-PE separators. These curves clearly illustrated their tensile strengths, measured at 46.1 MPa, 57.0 MPa, and 55.3 MPa, respectively. Through further data comparison, it became apparent that both α-Al_2_O_3_ and N-Al_2_O_3_ contributed to enhancing the strength of separators, which was attributed to the inherent rigidity of α-Al_2_O_3_ and N-Al_2_O_3_. In addition to dimensional stability and mechanical properties at high temperatures, porosity, electrolyte absorption capacity, and compatibility with the electrolyte are all crucial factors for evaluating the application effectiveness of separators. Figure 6b demonstrates that the N-Al_2_O_3_-PE separator excels in terms of porosity and electrolyte absorption capacity, achieving impressive levels of 50% and 150%, respectively. These results significantly surpassed those of the PE (44% and 80%) and α-Al_2_O_3_-PE (39% and 120%) separators. The superior performance of the N-Al_2_O_3_-PE separator can be attributed to the hydrophilic nature and unique internal structure of N-Al_2_O_3_. To accurately assess the wettability of the separators, water with a similar polarity as liquid electrolytes was applied to measure the wettability of the separators [5,27]. As shown in Figure 6c, compared to the PE separator, the α-Al_2_O_3_-PE and N-Al_2_O_3_-PE separators exhibited superior wettability. The improvement of the wettability of separators not only simplified the battery assembly process but also ensured the long-term stable preservation of the liquid electrolyte, thereby effectively extending the cycling life of the battery. Moreover, with the aim of further evaluating the compatibility between the separator and liquid electrolyte, the liquid electrolyte was dropped onto the surface of the separators (Figure 6d). Thanks to the existence of α-Al_2_O_3_ and N-Al_2_O_3_, liquid electrolytes dropped on the α-Al_2_O_3_-PE and N-Al_2_O_3_-PE separators were easily absorbed and spread on the separator. In contrast, the liquid electrolyte on the PE separators maintained its original hemispherical shape for an extended period.

**Figure 7:**
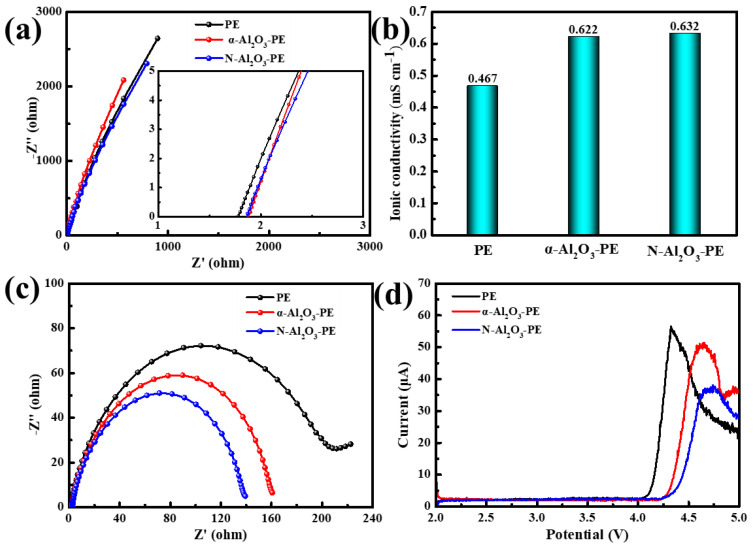
(**a**) Nyquist plots of the SS/SS batteries, (**b**) the corresponding ionic conductivity, (**c**) Nyquist plots of the Li/Li symmetrical batteries, and (**d**) LSV curves of Li/SS batteries with different separators.

The ionic conductivity is a vital indicator reflecting the rate of ion transport in LIB separators. Figure 7a presents data on the bulk resistance (Rb) of various separators. Based on the real intercept value shown in Figure 7a, the ionic conductivity of the separators can be accurately calculated using Formula (3). The magnitude of ionic conductivity primarily depends on the electrolyte absorption capacity and wettability of the separators. As presented in Figure 7b, compared to the PE separator with an ionic conductivity of 0.467 mS/cm, the ionic conductivity of α-Al_2_O_3_-PE and N-Al_2_O_3_-PE separators expanded to 0.622 and 0.632 mS/cm, respectively. In LIBs, the electrochemical stability of the battery heavily relies on the interfacial resistance between the separators and the electrodes. As vividly illustrated in Figure 7c, the Li/Li batteries equipped with N-Al_2_O_3_-PE separators exhibited notably lower interfacial impedance compared to those using PE or α-Al_2_O-PE separators, suggesting a superior interfacial compatibility between the N-Al_2_O_3_-PE separators and the electrodes. Such commendable performance can be credited to the high porosity, outstanding wettability, and exceptional electrolyte absorption capacity of the separators. Furthermore, Figure 7d illustrates the linear sweep voltammetry (LSV) curves for Li/SS batteries utilizing various separators across a voltage spectrum from 2.0 V to 5.0 V. Evidently, the N-Al_2_O_3_-PE separators demonstrated remarkable anode stability relative to Li^+^/Li, reaching up to 4.25 V, which was higher than that of the PE and α-Al_2_O_3_-PE separators. This enhancement in stability is primarily due to the superior ionic conductivity imparted by the N-Al_2_O_3_ coating, coupled with its effective suppression of lithium salt anion decomposition [28].

**Figure 8:**
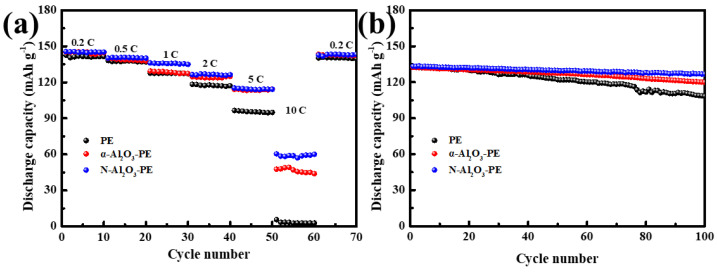
(**a**) The C–rate performance of LiCoO_2_/Li batteries with different separators and (**b**) cycle performance of LiCoO_2_/Li batteries with different separators at the current density of 1 C.

**Figure 9:**
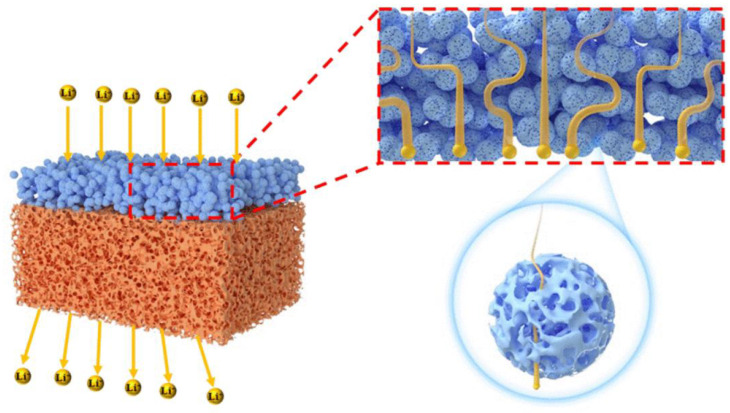
Schematic diagram of Li^+^ ions passing through the N-Al_2_O_3_-PE separators.

To further study the electrochemical performance of these separators, the rate and cycle performance of LiCoO_2_/Li half batteries with the PE, α-Al_2_O_3_-PE, and N-Al_2_O_3_-PE separators were tested at room temperature in the range of 3.0–4.2 V. It can be observed in Figure 8a that the discharge capacity of the batteries with different separators exhibited a declining trend with the growth of discharge rates. Among these separators, the discharge capacity of the PE separator batteries dropped the most severely and even lost discharge capacity at 10 C, which was only 5.7 mAh g^−1^. As for the α-Al_2_O_3_-PE and N-Al_2_O_3_-PE separators, they exhibited similar rate performances at 0.2, 0.5, 1, 2, and 5 C. However, at 10 C, the discharge capacity of the N-Al_2_O_3_-PE separators (60.4 mAh g^−1^) was significantly higher than that of the α-Al_2_O_3_-PE separators (47.6 mAh g^−1^). The significant difference was primarily attributed to the distinct characteristics of N-Al_2_O_3_, specifically its internal multi-dimensional network pore structure, which created an interconnected and efficient “highway” for Li^+^ ion transport, consequently enhancing the shuttle efficiency of lithium ions. Figure 9 illustrates the schematic diagram of Li^+^ ions passing through the N-Al_2_O_3_-PE separators. In addition to passing through the gap between N-Al_2_O_3_ particles, Li^+^ can also pass through the inside pores of N-Al_2_O_3_, which was the reason why the N-Al_2_O_3_-PE separators presented an excellent rate performance. Figure S2 shows the discharge capacity curves for the first cycle of the LiCoO_2_/Li battery equipped with the PE, α-Al_2_O_3_-PE, and N-Al_2_O_3_-PE separators at different discharge rates. The cycle performance of batteries with different separators at the current density of 1 C is illustrated in Figure 8b. More significantly, the battery with the N-Al_2_O_3_-PE separators processed a remarkable cycle stability with a discharge capacity of 126.8 mAh g^−1^ after 100 cycles at the current density of 1 C, and the capacity retention reached 95%, which was higher than that of the PE (82%) and α-Al_2_O_3_-PE (91%) separators. As is well known, the cycle performance depends on the solid electrolyte interphase (SEI) layer and the growth of the internal resistance of the batteries [14]. Firstly, the N-Al_2_O_3_-PE separators, with its excellent mechanical properties, ensures the stable existence of the SEI layer and effectively accommodates the stress arising from volume changes during the battery’s charging and discharging processes [29,30]. Secondly, the N-Al_2_O_3_-PE separators provided migration channels for Li^+^, helping to reduce the transport distance and obstruction of Li^+^ ions during the process of charging and discharging. Finally, the multi-dimensional network structure of N-Al_2_O_3_ can adsorb more electrolytes, reducing its loss during battery cycling, which helps maintain the electrolyte balance within the battery and has a positive effect on the improvement of battery cycling stability. The charge–discharge curves of LiCoO_2_/Li batteries with different separators at different cycles are shown in Figure S3.

**Figure 10:**
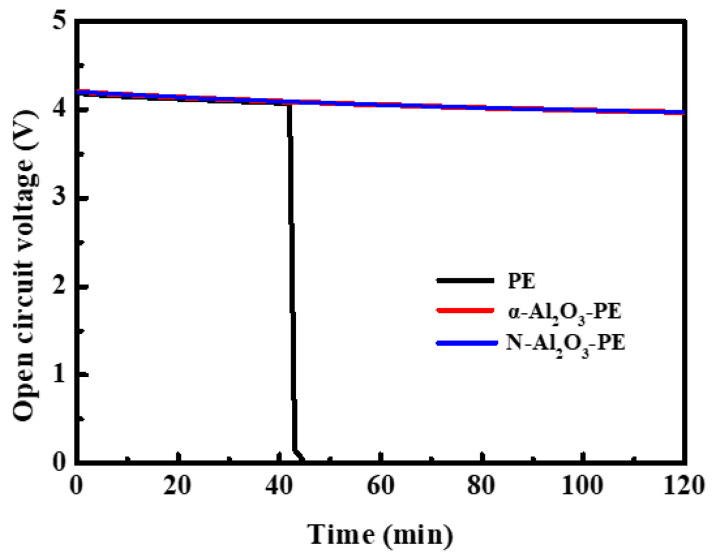
Open circuit voltage of LiCoO_2_/Li half batteries equipped with PE, α-Al_2_O_3_-PE, and N-Al_2_O_3_-PE separators at 140 °C.

To evaluate the thermal stability of the separators in the battery system, the open circuit voltage (OCV) of a LiCoO_2_/Li half battery equipped with PE, α-Al_2_O_3_-PE, and N-Al_2_O_3_-PE separators was continuously monitored at 140 °C for 120 min. As shown in Figure 10, the OCV of the battery equipped with the PE separators dropped sharply after 40 min, which was due to the thermal contraction of the PE separators, resulting in an internal short circuit of the battery. In contrast, the OCV curve of the α-Al_2_O_3_-PE and N-Al_2_O_3_-PE batteries presented only a slow decline throughout the measurement period, mainly originating from an increase in self-discharge at high temperatures. Overall, the N-Al_2_O_3_-PE separators with excellent thermal stability are beneficial to improve the safety of LIBs and is an ideal separator for LIBs.

## 4. Conclusions

In the current study, N-Al_2_O_3_ with a multi-dimensional network pore structure was fabricated. At the same time, the N-Al_2_O_3_-PE separators were prepared by coating N-Al_2_O_3_ on one side of the PE separators. The results indicated that the N-Al_2_O_3_-PE separators excelled in terms of thermal stability and affinity with electrolytes. Beyond that, the N-Al_2_O_3_-PE separators exhibited excellent ionic conductivity (0.632 mS cm^−1^) due to the internal multi-dimensional network pore structures of N-Al_2_O_3_, which provided an interconnected and efficient “highway” for the transportation of Li^+^ ions. When the N-Al_2_O_3_-PE separators were employed in LIBs, the LiCoO_2_/Li half batteries exhibited remarkable rate and cycle performance. Notably, at a current density of 10 C, the discharge capacity of batteries with N-Al_2_O_3_-PE separators reached an impressive 60.4 mAh g^−1^, whereas the batteries equipped with PE separators managed only 5.7 mAh g^−1^. We anticipate that the N-Al_2_O_3_-PE separators will pave the way for the development of high-performance LIBs with enhanced rate capabilities and cycling stability in future applications.

## Figures and Tables

**Table 1:** The average particle size of the as-obtained sample.

Sample	S0.1–8	S0.2–8	S0.3–8	S0.5–8
Average size (μm)	4.75 ± 1.64	4.50 ± 1.37	3.07 ± 0.78	2.64 ± 0.64

## Data Availability

The original contributions presented in this study are included in the article and Supplementary Material. Further inquiries can be directed to the corresponding authors.

## Supplementary Materials

The following supporting information can be downloaded at: https://www.mdpi.com/article/10.3390/ma18030660/s1, Figure S1: The average particle size of the as-sample (a) S0.1–8, (b) S0.2–8, (c) S0.3–8, (d) S0.5–8; Figure S2: The discharge capacity curves for every first cycle of each discharge rate of (a) PE, (b)α-Al_2_O_3_-PE, and (c) N-Al_2_O_3_-PE separators; Figure S3: The charge-discharge curves of LiCoO_2_/Li batteries with different separators at different cycle (a) 1st, (b) 10th, (c) 50th, and (d) 100th.
