# Supplementary material for: Fabrication of Network Spherical α-Al2O3 and Its Application on the Separator of Lithium-Ion Batteries
Source: Materials (Basel). 2025 Feb 2;18(3):660. doi: 10.3390/ma18030660 (PMC11820654; doi:10.3390/ma18030660)
Supplement: Supplementary file 1 [file materials-18-00660-s001.zip › materials-3438216-supplementary.pdf]

Supplementary materials

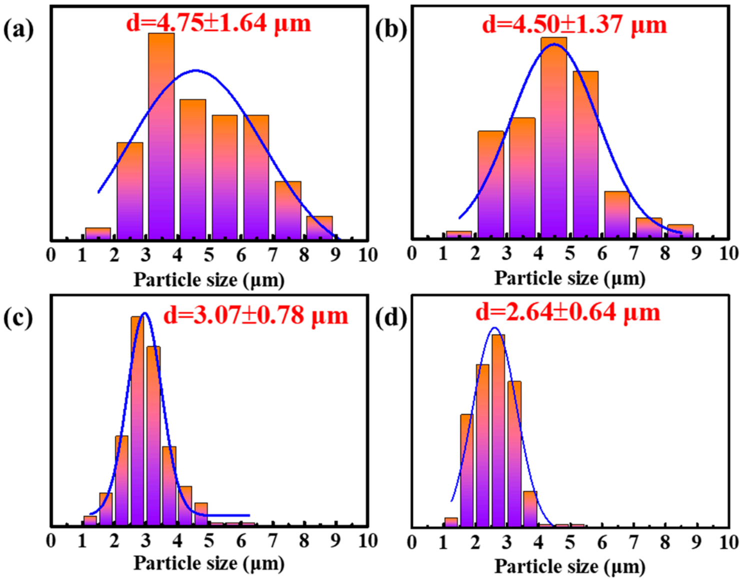

Fig. S1. The average particle size of the as-sample (a) S0.1-8, (b) S0.2-8, (c) S0.3-8, (d) S0.5-8

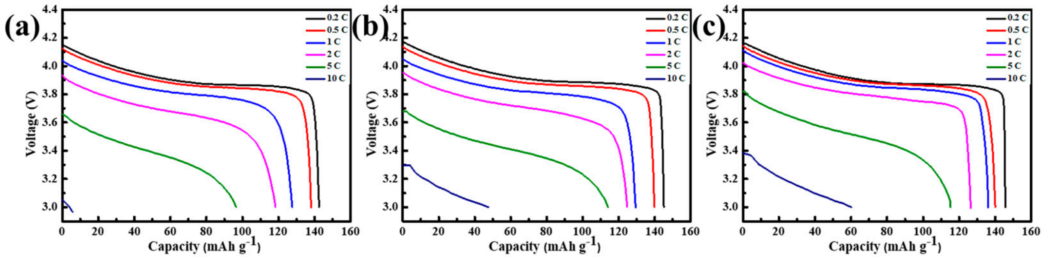

Fig. S2. The discharge capacity curves for every first cycle of each discharge rate of (a) PE, (b)α-Al_2O_3-PE, and (c) N-Al_2O_3-PE separators

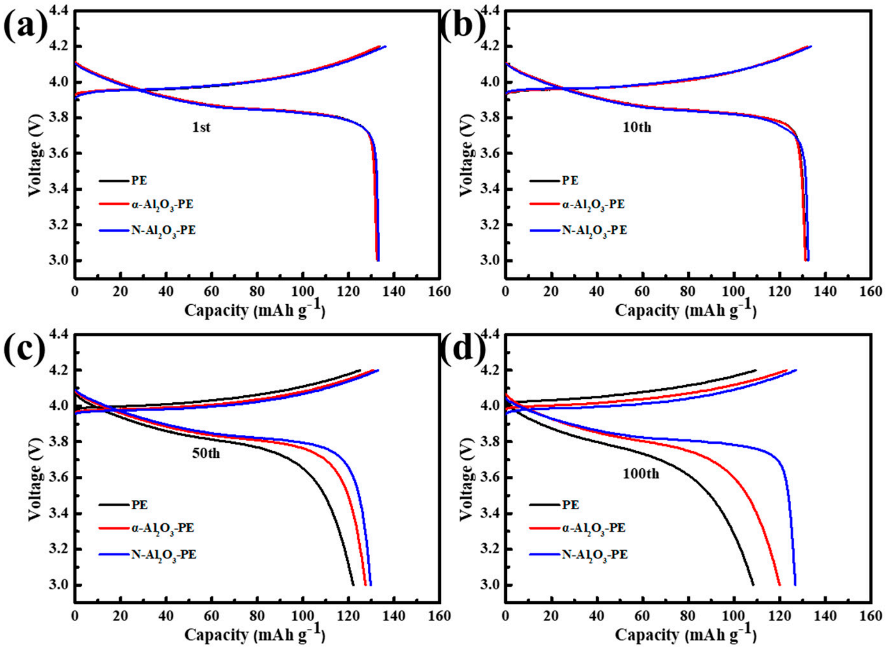

Fig. S3. The charge-discharge curves of $LiCoO_2$/Li batteries with different separators at different

cycle (a) 1st, (b) 10th, (c) 50th, and (d) 100th
